# Surface Morphology-Dependent Functionality of Titanium Dioxide–Nickel Oxide Nanocomposite Semiconductors

**DOI:** 10.3390/nano9121651

**Published:** 2019-11-21

**Authors:** Yuan-Chang Liang, Nian-Cih Xu, Kai-Jen Chiang

**Affiliations:** Department of Optoelectronics and Materials Technology, National Taiwan Ocean University, Keelung 20224, Taiwan; sad821008@gmail.com (N.-C.X.); ssdcjh972003@yahoo.com.tw (K.-J.C.)

**Keywords:** surface, morphology, semconductors, functionality

## Abstract

In this study, TiO_2_–NiO heterostructures were synthesized by combining hydrothermal and chemical bath deposition methods. The post-annealing temperature was varied to control the surface features of the TiO_2_–NiO heterostructures. TiO_2_–NiO heterostructures annealed at 350 °C comprised NiO-nanosheet-decorated TiO_2_ nanostructures (NST), whereas those annealed at 500 °C comprised NiO-nanoparticle-decorated TiO_2_ nanostructures (NPT). The NPT exhibited higher photodegradation activity than the NST in terms of methylene blue (MB) degradation under irradiation. Structural analyses demonstrated that the NPT had a higher surface adsorption capability for MB dyes and superior light-harvesting ability; thus, they exhibited greater photodegradation ability toward MB dyes. In addition, the NST showed high gas-sensing responses compared with the NPT when exposed to acetone vapor. This result was attributable to the higher number of oxygen-deficient regions on the surfaces of the NST, which increased the amount of surface-chemisorbed oxygen species. This resulted in a relatively large resistance variation for the NST when exposed to acetone vapor.

## 1. Introduction

TiO_2_ has been widely studied for applications in photodegradation organic dyes and gas-sensing materials because of its low toxicity, high chemical stability, and environmental friendliness [1,2,3,4,5,6]. However, TiO_2_ still has some drawbacks that might limit its efficiency in photocatalyst and gas-sensing materials. The wide band gap of TiO_2_ limits its ability to absorb visible light; moreover, rapid recombination of photogenerated electron–hole pairs hinders its performance in photodegrading organic dyes [7]. TiO_2_ has been favored for sensing H_2_, NO_2_, and C_2_H_5_OH at elevated temperatures of 290–550 °C [8]; nevertheless, pure TiO_2_ with improved gas-sensing response is still highly desired for fabricating gas sensors with high gas detection performance. Recently, constructing TiO_2_-based heterostructures proved to be a promising approach to enhancing the photodegradation and gas-sensing abilities of TiO_2_. Several TiO_2_-based heterostructure systems have been proposed and investigated. Decorating the surface of TiO_2_ with a CdS photosensitizer improves its photodegradation ability toward rhodamine B [9]. SrTiO_3_/TiO_2_ heterostructures are beneficial for the rapid separation of photogenerated electrons and holes, and they improve the photodegradation performance of pristine TiO_2_ [10]. TiO_2_–ZnO composite tubes exhibit higher organic dye photodegradation ability when compared with pristine TiO_2_ tubes [11]. According to research fabricating TiO_2_-based heterostructures in applications such as gas-sensing materials, ZnS-sphere-decorated TiO_2_ flower-like composites exhibit a superior gas-sensing response to pristine TiO_2_ flowers [3]. Moreover, decorating the surface of TiO_2_ nanorods with island-like CdO crystallites markedly improves the gas-sensing response of the nanorods to low-concentration NO_2_ [2]. Increased potential barriers in the heterostructure systems could constitute an essential factor in enhancing the gas-sensing performance of TiO_2_ on exposure to reducing and oxidizing gases. Accordingly, most proposed TiO_2_-based heterostructures are based on n-type oxides or sulfides uniformly distributed on the surfaces of n-type TiO_2_, leading to the formation of n–n-type heterogeneous structures in the composite systems.

However, in addition to n–n-type TiO_2_-based heterogeneous systems, recent efforts have focused on improving the photodegradation and gas-sensing performance of TiO_2_ with a p–n-type heterogeneous system. NiO is a p-type metal-oxide semiconductor with a wide band-gap energy (Eg = 3.6–4.0 eV) [12]. Creating TiO_2_–NiO p–n heterojunctions is one of the most effective strategies for developing efficient TiO_2_-based photocatalysts. When p-type NiO and n-type TiO_2_ form a p–n junction, an inner electric field is established at the interface, which then acts as a potential barrier to prevent the recombination of electrons and holes. Therefore, improved photodegradation performance towards organic dyes is expected for TiO_2_–NiO p–n-type heterostructures [13]. Moreover, p-type NiO is a suitable catalyst for promoting selective oxidation of various volatile organic compounds [14]. Constructing TiO_2_–NiO heterostructures is also a promising approach to improving both gas-sensing abilities of n-type TiO_2_ [15]. However, systematic studies on NiO loading effects on both the photodegradation and gas-sensing properties of TiO_2_ are still limited in number. In the current study, TiO_2_ flower-based heterostructures were synthesized and decorated with various NiO shell layer crystallites. The three-dimensional (3D) architecture of TiO_2_ nanostructures were used as templates to fabricating TiO_2_–NiO nanocomposites herein. This is due to the fact that 3D nanotechnology of oxides has been demonstrated to obtain remarkable properties and multifunctionality [16]. Controlling the post-annealing procedure resulted in two types of TiO_2_–NiO composites—namely NiO nanosheet-decorated TiO_2_ flowers and NiO nanoparticle-decorated TiO_2_ nanostructures—that were used to understand the effects of different morphologies and microstructures on photodegradation ability and gas-sensing responses. This study proposes a correlation between the microstructures of the NiO shell layer and the photodegradation and gas-sensing performance of the TiO_2_–NiO composites. The investigations herein are important for designing TiO_2_–NiO nanocomposites in photodegrading organic dyes and gas-sensing applications with satisfactory performance.

## 2. Materials and Methods

In this study, the TiO_2_ nanostructures were grown on glass substrates using a hydrothermal method. For hydrothermal synthesis, 2.4 mL of deionized water was mixed with 3.6 mL of concentrated HCl (35%) in a Teflon-lined stainless autoclave. The mixture was stirred at ambient conditions for 5 min and after that, 0.25 mL of TiCl_4_ was added into the mixed solution for preparation of TiO_2_ nanostructures. The hydrothermal synthesis was conducted at 180 °C for 3 h. NiO crystallites were decorated onto the surfaces of TiO_2_ nanostructures by a chemical bath deposition (CBD) method. The solution for CBD process was prepared by adding 1 mL of aqueous ammonia (25–28%) to the mixture of 20 mL of 1 M nickel sulfate and 16 mL of 0.25 M potassium persulfate. The TiO_2_ nanostructures were immersed into the CBD solution for reaction and the samples were rinsed with distilled water. Finally, the as-synthesized samples were post-annealed at 350 and 500 °C in ambient air for 1 h to fabricate the TiO_2_–NiO heterostructures with various surface crystal features. Notably, the market crystalline temperature of the NiO phase from initially formed Ni-based hydroxide nanosheets was above 350 °C in this study. The sample annealed at 350 °C formed NiO-nanosheet-decorated TiO_2_ nanostructures (NST); that annealed at 500 °C formed NiO-nanoparticle-decorated TiO_2_ nanostructures (NPT). The NST and NPT were used to present the corresponding samples annealed at 350 and 500 °C, respectively.

Crystal structures of the as-synthesized samples were investigated by X-ray diffraction (XRD) using Cu Kα radiation. The scanning electron microscopy (SEM) was performed to investigate surface morphology of the samples. High-resolution transmission electron microscopy (HRTEM) was used to investigate the detailed microstructures of the TiO_2_–NiO composites. The attached energy dispersive X-ray spectroscopy (EDS) was used to investigate the compositions of the TiO_2_–NiO composite samples. X-ray photoelectron spectrometry (XPS) analysis was used to determine the chemical binding status of constituent elements of the samples. The analysis of absorbance spectra of the composites was conducted by using a UV–Vis spectrophotometer. Photodegradation performance of various TiO_2_–NiO composites were performed by comparing the degradation of aqueous solution of methylene blue (MB, 10^−6^ M) containing various TiO_2_–NiO composites as catalysts under solar light irradiation excited from a 100 W Xe arc lamp. The variation of MB solution concentration in the presence of various TiO_2_–NiO composites with different irradiation durations was analyzed by recording the changes of absorbance spectra using a UV–Vis spectrophotometer. The gas-sensing response of the gas sensors made from the TiO_2_–NiO composites to acetone vapor with concentrations of 50–750 ppm was defined as the Rg/Ra. Ra was the sensor electrical resistance in the absence of the target gas and Rg was that in the target gas.

## 3. Results and Discussion

**Figure 1:**
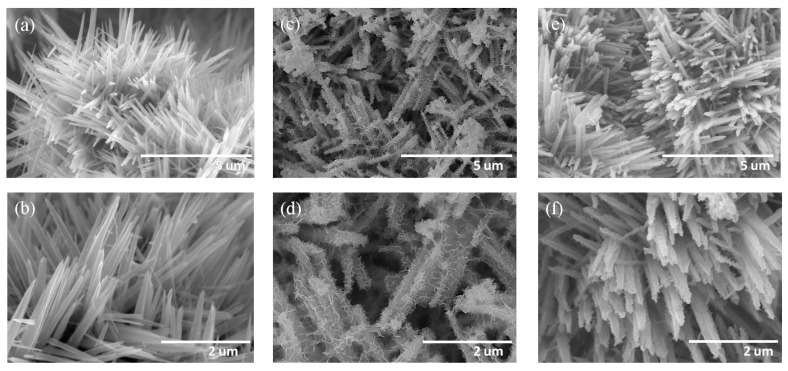
(**a**,**b**) Low- and high-magnification SEM images of TiO_2_ nanostructures. (**c**,**d**) Low- and high-magnification SEM images of TiO_2_–NiO composites formed at 350 °C. (**e**,**f**) Low- and high-magnification SEM images of TiO_2_–NiO composites formed at 500 °C.

**Figure 2:**
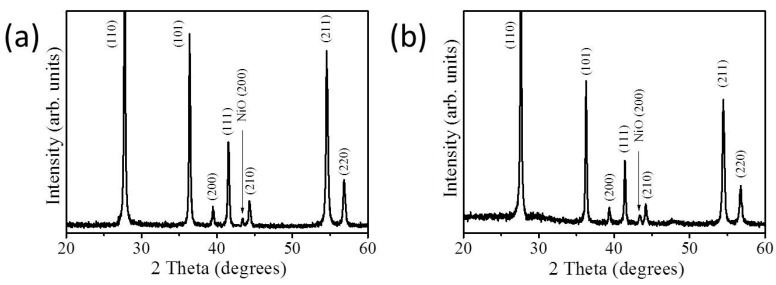
XRD patterns of TiO_2_–NiO composites formed at (**a**) 350 °C and (**b**) 500 °C. The arrow represents the position of NiO Bragg reflection.

Figure 1a,b depict the SEM images of hydrothermally derived 3D TiO_2_ nanostructures. The lengths of the TiO_2_ petals ranged from 1.2 to 3.1 μm and the surface of TiO_2_ was smooth. The possible growth mechanism of the hydrothermally derived TiO_2_ nanostructures herein has been reported elsewhere [3]. Figure 1c,d illustrate the SEM images of TiO_2_–NiO composites formed through post-annealing at 350 °C. The surfaces of the TiO_2_–NiO composites consisted of undulated shell layers and were rough. The coverage of the sheet-like NiO crystallites on the surfaces of TiO_2_ nanostructures was uniform, and a homogeneous layer-like structure was thus formed. The sheet thickness of the NiO crystallites ranged from 10 to 25 nm. When the post-annealing temperature was further increased to 500 °C, the morphology of the NiO crystallites decorated on the surfaces of the TiO_2_ structures markedly differed from that observed at 350 °C (Figure 1e,f). Comparatively, the surface morphology of the NiO thin layer on the TiO_2_ surface became a discontinuous shell structure when the TiO_2_–NiO composites were formed at 500 °C. According to the SEM observations, the morphology of TiO_2_–NiO composites can be controlled by changing the post-annealing temperature. Figure 2a,b illustrate the XRD patterns of the TiO_2_-based composites with various thermal post-annealing procedures. Several sharp and intense Bragg reflections originating from the rutile TiO_2_ phase (JCPDS No. 004−0551) were observed (Figure 2a,b). The TiO_2_ nanostructures were highly crystalline, as revealed by the XRD patterns. In addition to the Bragg reflections originating from the TiO_2_ phase (Figure 2a,b), a tiny Bragg reflection centered at approximately 43.4° was observed and was ascribed to the (200) plane of the cubic NiO structure (JCPDS No. 02−1216) [17]. No Bragg reflections from other impurities were detected. The SEM and XRD results demonstrated that crystalline TiO_2_–NiO composites with various NiO shell layer morphologies could be formed through various post-annealing procedures.

**Figure 3:**
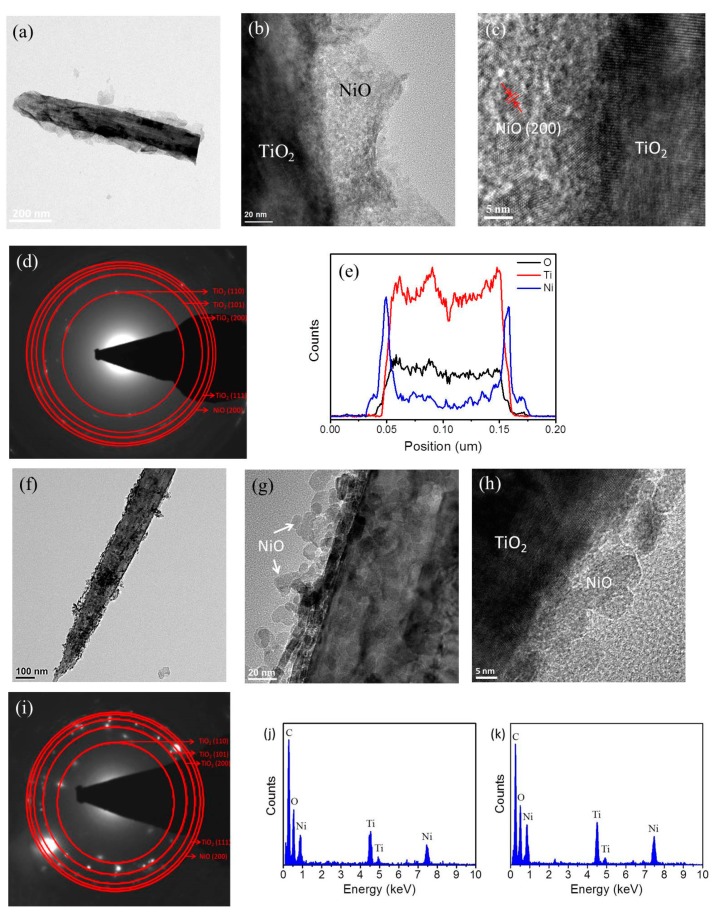
TEM analyses of TiO_2_–NiO composites formed at 350 °C: (**a**) Low-magnification TEM image of a single TiO_2_–NiO composite. (**b**) High-magnification TEM image taken from the TiO_2_–NiO composite. (**c**) High-resolution transmission electron microscopy (HRTEM) image taken from the local region of the TiO_2_–NiO composite. (**d**) Selected area electron diffraction (SAED) pattern of several TiO_2_–NiO composites. (**e**) Energy dispersive X-ray spectroscopy (EDS) line-scanning profiles of the TiO_2_–NiO composite. TEM analyses of TiO_2_–NiO composites formed at 500 °C. (**f**,**g**) Low- and high-magnification TEM images of a single TiO_2_–NiO composite, respectively. (**h**) HRTEM image taken from the local region of the TiO_2_–NiO composite. (**i**) SAED pattern of several TiO_2_–NiO composites. (**j**,**k**) EDS spectra of the NiO-nanoparticle-decorated TiO_2_ nanostructures (NPT) and NiO-nanosheet-decorated TiO_2_ nanostructures (NST) composites, respectively.

Figure 3a displays the low-magnification TEM image of a single TiO_2_–NiO composite formed at 350 °C. For a representative core–shell structure, a single TiO_2_ petal was uniformly covered with interconnected thin NiO sheets when the as-synthesized composite was annealed at 350 °C. Figure 3b shows distinct boundaries between the adjacent sheet-like NiO crystallites. As revealed by the HRTEM image depicted in Figure 3c, lattice fringes with multiple orientations were observed in the NiO crystallites, revealing a polycrystalline feature. Moreover, lattice fringes with an interval of 0.20 ± 0.02 nm were determined to correspond to the interplanar distance of NiO (200). Selected area electron diffraction (SAED) patterns of several NST composites were recorded, as shown in Figure 3d. The patterns exhibited several distinct diffraction rings consisting of sharp and bright spots that originated from the TiO_2_ petals and NiO sheets (Figure 3d). According to the SAED patterns, the (110), (101), (200), (111), (210), and (211) crystallographic planes were determined to correspond to the rutile TiO_2_ phase and the (200) crystallographic plane was determined to correspond to the cubic NiO phase. EDS line-scan analysis (Figure 3e) confirmed the localization of Ti in the core area and the tendency of the Ni element to be distributed along the surface of the TiO_2_ petal, revealing that the NiO sheets adequately covered the TiO_2_ surface. By contrast, the TEM images in Figure 3f,g reveal the morphology of the TiO_2_–NiO composite formed at 500 °C to differ from that of the composite annealed at 350 °C. NiO crystallites with a particle-like feature discontinuously decorated the surface of the TiO_2_ petal. As demonstrated by the HRTEM image in Figure 3h, the NiO particles had a diameter of 10 to 18 nm. The NPT formed at 500 °C exhibited appropriate crystalline phases (Figure 3i). The corresponding EDS analyses (Figure 3j,k) demonstrated that Ti, Ni, and O were the main constituent elements of the composites; moreover, no impurity atoms were detected. The Ni/Ti wt% ratios of the NPT and NST were 0.68 and 0.73, respectively in this study.

**Figure 4:**
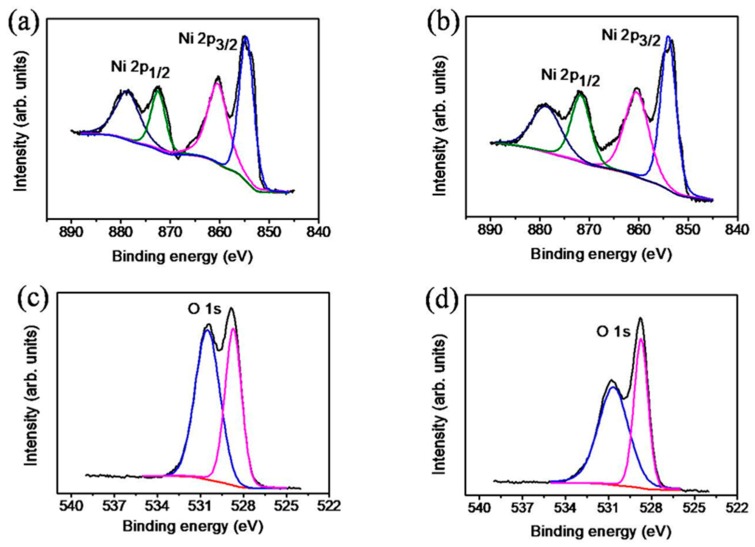
High-resolution XPS spectra in Ni 2p region of TiO_2_–NiO composites formed at various temperatures: (**a**) 350 °C and (**b**) 500 °C. High-resolution XPS spectra in O 1s region of TiO_2_–NiO composites formed at various temperatures: (**c**) 350 °C and (**d**) 500 °C.

Figure 4a,b show the XPS narrow-scan spectra of the Ni 2p region of the TiO_2_–NiO composites formed at 350 °C and 500 °C, respectively. The Ni 2p states of the NiO shell layers (Figure 4a,b) contained multiple peaks at binding energies between 854 and 879 eV, including main peaks and satellites. The main peaks of Ni 2p_3/2_ and Ni 2p_1/2_ were located at approximately 854.1 and 871.8 eV, respectively (Figure 4a,b), which were in agreement with those reported in the literature for the NiO phase [18,19]. Figure 4c,d show the O1s spectra of the TiO_2_–NiO composites formed at 350 °C and 500 °C, respectively. Sharp fitted subpeaks were observed at a binding energy of approximately 528.8 eV and were attributed to the lattice oxygen of the NiO phase [20]; additionally, broadened subpeaks were observed at 530.7 eV and were attributed to the formation of surface-oxygen-deficient sites and surface chemisorbed oxygen species on the NiO shell layer [21]. Notably, the relative intensity of the higher binding energy component in the O1s spectra of the TiO_2_–NiO composites decreased when the composites were thermally treated from 350 °C to 500 °C. This finding reveals that surface crystalline imperfections of a larger size were formed in the NiO nanosheets.

**Figure 5:**
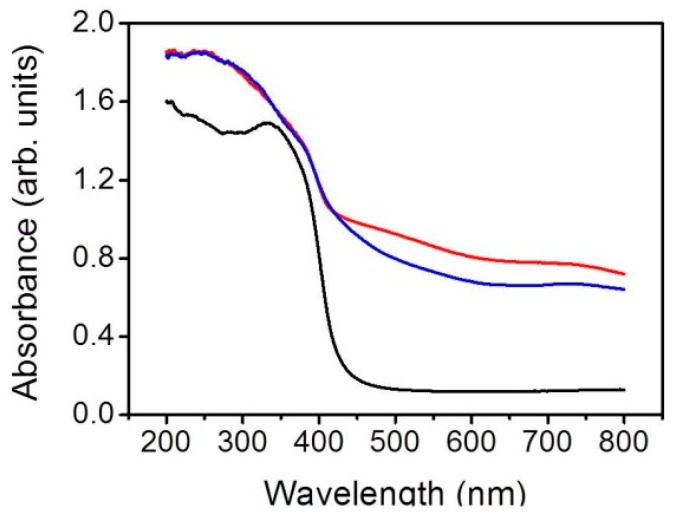
Optical absorbance spectra of the pristine TiO_2_ (black), NST (blue), and NPT (red).

Figure 5 presents the optical absorption properties of the pristine TiO_2_, NST, and NPT. For the TiO_2_, a sharp drop of the absorption edge was observed, and the onset was at approximately 406 nm, which is consistent with the intrinsic band-gap absorption of rutile TiO_2_ [22]. After the NiO nanosheets and nanoparticles were coated onto the surfaces of the TiO_2_ nanostructures, the absorption edge broadened, and a redshift extension in the absorption edge was observed. The extended absorption edge of the TiO_2_–NiO composites suggests strong contact between NiO and TiO_2_ engendered by the interdispersion of two semiconductors [7]. In the interface region, the overlap of the conduction band due to the Ti d orbital of titanium oxide and Ni d orbital of nickel oxide may reduce the energy gap between the Ti d and O p orbitals of titanium oxide and enable a redshift of the adsorption edge [23]. Comparatively, the NPT exhibited a higher light harvesting ability. The extension of the optical absorption edge to the visible light region of the TiO_2_–NiO composites can enhance the efficiency of forming electron–hole pairs on the composite photocatalyst surfaces under illumination and might improve the photodegradation performance of the TiO_2_ nanostructures [7]. A similar redshift of TiO_2_ adsorption edge because of the surface decoration of NiO crystals has been shown in other TiO_2_–NiO composites synthesized via sol–gel and electroplating methods [24,25].

**Figure 6:**
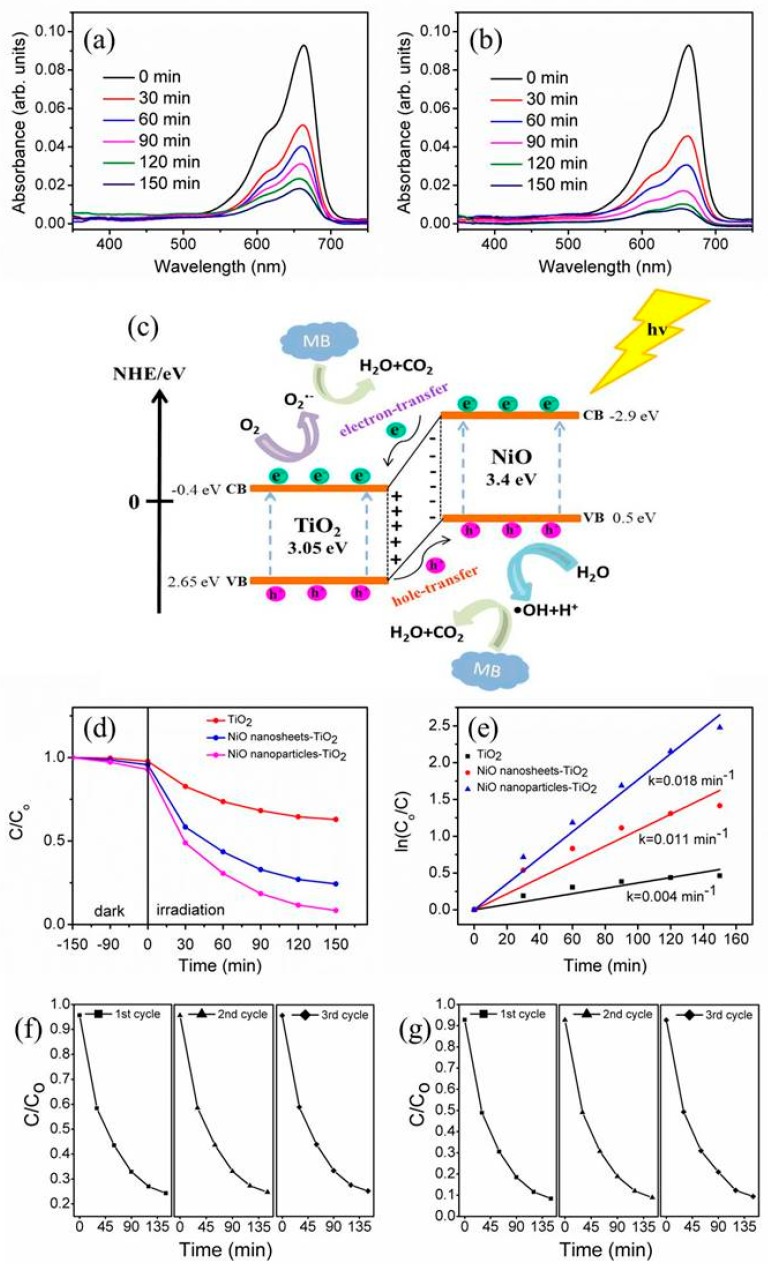
Intensity variation of absorbance spectra of the methylene blue (MB) solution vs. irradiation duration containing various TiO_2_–NiO composites under solar light irradiation: (**a**) NST. (**b**) NPT. (**c**) Schematic of photodegradation process of TiO_2_–NiO composites toward MB. (**d**) The ratio of the remaining MB concentration after light irradiation (C) and the initial MB concentration without light irradiation (C_o_) vs. irradiation time curves for the MB solution containing various TiO_2_–NiO composites in dark conditions and under light irradiation. (**e**) Plot of ln (C_o_/C) vs. reaction time for MB solution containing various TiO_2_–NiO composites under irradiation. (**f**,**g**) Recycled performances of photodegradation of MB solution in the presence of the NST and NPT, respectively.

The photodegradation performance of the various TiO_2_–NiO composites was examined in terms of MB solution degradation. This study monitored the variation of the characteristic absorption intensity (at approximately 663 nm) of the MB solution containing various TiO_2_–NiO composites with different irradiation durations, as illustrated in Figure 6a,b. The absorbance spectra of the MB solution at 663 nm decreased with irradiation duration, indicating that the MB dyes were efficiently degraded by the TiO_2_–NiO photocatalysts under irradiation. The photodegradation reaction of the MB solution containing TiO_2_–NiO composites involved the reaction of photoexcited free electrons (TiO_2_-core) and holes (NiO-shell) with the MB solution. This can be understood from the possible band alignment between the TiO_2_ and NiO reported previously [26,27]. The subsequently formed hydroxyl radicals in the solution were strong oxidizing agents that effectively decomposed MB dyes [28,29]. The schematic figure of the photodegradation reaction of TiO_2_–NiO towards MB solution is illustrated in Figure 6c. The absorption intensity of the MB solution containing the NPT decreased more rapidly than that of the MB solution containing the NST under the given irradiation duration. Furthermore, the C/C_o_ ratio was used to determine the photodegradation level of the MB solution containing various composite photocatalysts, where C represents the remaining MB concentration after light irradiation and C_o_ represents the initial MB concentration without light irradiation [30]. A plot of C/C_o_ versus irradiation duration is presented in Figure 6d. For comparison, the photodegradation level of the MB solution containing various composites under dark conditions is also shown in Figure 6d. Notably, the C/C_o_ values for the degradation of the MB solution under dark conditions for 150 min were 7.9% and 4.3% for the NPT and NST, respectively. This reveals that the NPT possessed a higher MB dye surface adsorption capability. The relatively superior adsorption capability of the NPT enabled the MB molecules to diffuse freely inside the composites and facilitated more efficient contact between the photocatalysts and organic dyes, thus improving the resulting photodegradation level [27]. The photodegradation efficiency observed for the TiO_2_–NiO composites was substantially superior to that observed for the pristine TiO_2_. Contact between p-NiO and n-TiO_2_ in the composites engendered an inner electric field. The as-formed inner field further caused the photoexcited holes to flow into the negative field and the photoexcited electrons to move to the positive field in the oxides. Therefore, the photoexcited electron–hole pairs were separated more effectively by the p–n junction formed in the TiO_2_–NiO composites than that formed in the pristine TiO_2_. This enabled the formation of more free carriers on the active sides of the composites and increased the photodegradation activity of the composites [31]. In addition, the NPT showed a higher degree of photodegradation than that of the NST. This study also applied the following formula to investigate the kinetics of the degradation of the MB solution containing various photocatalysts under light irradiation (Figure 6e): ln (C_o_/C) = kt, where k is the apparent reaction rate constant and t is the irradiation duration [32]. Figure 6e shows a linear relationship between ln (C_o_/C) and irradiation duration. A higher degradation reaction rate was observed for the MB solution containing the NPT. The superior light-harvesting ability of the NPT accounted for the difference in photodegradation ability between the NPT and NST. The photodegradation stability of the TiO_2_–NiO composites under irradiation in the MB solution was evaluated after the recycling of the composites (Figure 6f,g). After three test cycles, the NST and NPT maintained relatively consistent activity without apparent deactivation.

**Figure 7:**
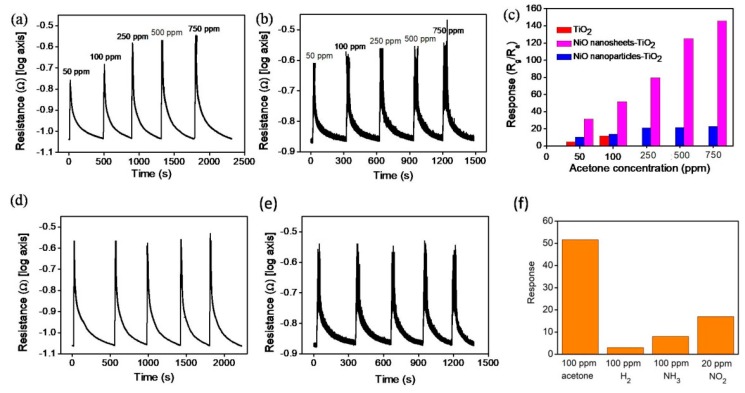
Gas-sensing response curves of various TiO_2_–NiO composites on exposure to various acetone vapor concentrations (50–750 ppm): (**a**) NST. (**b**) NPT. (**c**) Gas-sensing responses vs. acetone vapor concentrations for various TiO_2_–NiO composites. The gas sensing responses of the pristine TiO_2_ flowers to 50 and 100 ppm acetone vapor are also shown for a comparison. (**d**,**e**) Cyclic gas-sensing response curves of the NST and NPT on exposure to 250 ppm acetone vapor, respectively. (**f**) Gas-sensing selectivity of NST.

Figure 7a,b present the gas response transients of the NST and NPT, respectively, toward 50, 100, 250, 500, and 750 ppm acetone vapor at 300 °C. The gas-sensing response of the various composites increased with the acetone vapor concentration. As shown in Figure 7c, the gas-sensing response of the sensor comprising the NST increased from 31.3 to 145.5 when the acetone vapor concentration was increased from 50 to 750 ppm. By contrast, the gas-sensing responses of the sensor comprising the NPT were 10.2 and 22.8 with exposure to 50 and 750 ppm acetone vapor, respectively. The gas-sensing responses of the sensor comprising the NST were higher than those of the NPT under the given test conditions. Figure 7d,e show the results obtained from cyclic gas-sensing response tests of various TiO_2_–NiO composites on exposure to 250 ppm acetone vapor. The TiO_2_–NiO composites exhibited stability and reproducibility after five test cycles. For comparison, the gas-sensing responses of the pristine TiO_2_ exposed to 50 and 100 ppm acetone vapor are also displayed in Figure 7c. Decorating the surface of the TiO_2_ with NiO crystallites markedly improved the gas-sensing performance of the TiO_2_. For the pristine TiO_2_, the gas-sensing mechanism was explained mainly in terms of modulation of the depletion layer accompanying the adsorption and desorption of acetone molecules. When pristine TiO_2_ is exposed to air, oxygen molecules are adsorbed onto the surface of the TiO_2_ and are ionized to O^−^ by capturing free electrons from the conduction band of TiO_2_. This reduces the electron concentration, which then leads to the formation of an electron depletion layer. When TiO_2_ is exposed to acetone vapor, the acetone molecules react with the surface-adsorbed oxygen species of TiO_2_ according to the following equation [33]: CH_3_COCH_3(ads)_ + 8O^−^_(ads)_ = 3CO_2_ + 3H_2_O + 8e^−^. This reaction releases the trapped electrons back to the conduction band of TiO_2_, which increases the free-electron concentration, and ultimately reduces the resistance of the pristine TiO_2_. Notably, compared with the n-type gas-sensing behavior of pristine TiO_2_, the gas-sensing behavior of TiO_2_–NiO composites changes to p-type due to the p-type nature of NiO shells. In p–n oxide composites having a homogeneous p-type shell layer coverage, the gas-sensing behavior is dominated by p-type shell layers [6,34]. The possible difference in the gas-sensing ability of the NST and NPT might be associated with surface defect density differences. The variations of both the surface and interfacial potential barriers of the composites were determined to dominate the gas-sensing behavior of the TiO_2_–NiO_2_ composites with various shell layer morphologies. Defect analyses revealed that the NiO nanosheets contained more surface crystal point defects than those of the NiO nanoparticles. An increase in oxygen-deficient regions in oxides can be contributed to more surface-chemisorbed oxygen species being able to participate in the oxidation–reduction reaction occurring on the surface of the sensing materials, which would thus induce a greater change in sensor resistance [21]. A higher number of oxygen-deficient regions on the surfaces of NST would increase the amount of surface-chemisorbed oxygen species. In ZnO–ZnCr_2_O_4_ composites, a higher surface point defect density is highly associated with a higher sensing response toward ethanol vapor [35]. The structural analyses indicated that the NiO shell thicknesses of the composites were in the tens of nanometers; therefore, the interfacial potential barriers were assumed to be influenced by the surface adsorption and desorption of reductive acetone molecules. A larger potential barrier variation was expected in the NST than in the NPT during the gas-sensing tests. This explains the higher gas-sensing response of the NST in this study [2,36]. Moreover, the selectivity of the NST was shown in Figure 7f. A visible sensing response to acetone vapor of the NST was observed. Table 1 summarizes acetone vapor sensing responses of various TiO_2_–NiO composites reported in literatures for a comparison [8,37,38]. Notably, several reference works need a high operating temperature of 400 °C to obtain a visible gas-sensing response of TiO_2_–NiO composites. Comparatively, the NST herein exhibited superior acetone vapor sensing response and a relatively lower operating temperature.

## 4. Conclusions

TiO_2_–NiO heterostructures were successfully synthesized by combining a hydrothermal process and a chemical bath deposition method. Crystalline TiO_2_–NiO heterostructures were formed after post-annealing procedures, and the surface features of the TiO_2_–NiO heterostructures were controlled by varying the post-annealing temperature. Photocatalytic activity tests revealed that the NPT had a higher surface adsorption capability for MB dyes and light harvesting ability when compared with the NST. These factors accounted for the superior photocatalytic activity of the NPT. By contrast, gas sensors made from the TiO_2_–NiO heterostructures exhibited strong gas-sensing responses and recycling stability to acetone vapor. The NST exhibited a higher gas-sensing response than that of the NPT when exposed to acetone vapor under the given test conditions. The NiO nanosheets comprised more surface crystal point defects than the NiO nanoparticles did, which engendered the difference in potential barrier variation in the TiO_2_–NiO heterostructures with various surface morphologies during gas-sensing tests.

## Figures and Tables

**Table 1:** Comparison of acetone vapor sensing responses of various TiO_2_–NiO composites [8,37,38].

Materials	Operating Temperature	Acetone Concentration	Response (R_a_/R_g_ or R_g_/R_a_)	Reference
NiO nanosheets–TiO_2_ flowers	300 °C	100 ppm	51.6 (R_g_/R_a_)	(this work)
NiO nanoparticles–TiO_2_ flowers	300 °C	100 ppm	13.54 (R_g_/R_a_)	(this work)
TiO_2_–NiO nanorod	400 °C	200 ppm	9.81 (R_g_/R_a_)	[37]
TiO_2_–NiO nanorod	400 °C	200 ppm	9.33 (R_a_/R_g_)	[8]
95 wt% TiO_2_–5 wt% NiO composite film	300 °C	100 ppm	6 (R_g_/R_a_)	[38]
99 wt% TiO_2_–1 wt% NiO composite film	400 °C	100 ppm	6.5 (R_g_/R_a_)	[38]
